# Maternity Service in Bristol

**Published:** 1952-04

**Authors:** O. E. L. Sampson


### *Correspondence*

### Maternity Services in Bristol

Sir,

Professor Lennon's suggestions for a Maternity Service in Bristol present the general-practitioner obstetrician with a very gloomy future! The role he will have to play is so completely subordinate to the consultant, or his registrar that far from being an important member of the team, the G.P. is likely to become a " non-travelling reserve ".

The scheme proposed by the Bristol Local Medical Committee is one which visualises, the G.P. obstetrician as the mainstay of the service, backed by easily available and efficient consultant and emergency services. Professor Lennon's proposals reverse this idea and remove so much of the G.P's responsibility in the case that his pleasure in the work, and all incentives to improve his standards, will be destroyed.

I believe Professor Lennon is right when he says that domiciliary midwifery will become a thing of the past. The existence of the G.P. obstetrician will then depend on the type of obstetrical hospital that is built. If it takes the form of a relatively small institution serving a locality, then the G.P. obstetrician will be able to work under good and stimulating conditions and will flourish. But if the trend is towards massive centralized obstetrical units, then the G.P. will perish beneath a mass of consultants, registrars, obstetric house surgeons and midwives, the last three clamouring to do the work that is being " supervised " by the first.

O. E. L. Sampson

